# Hypoglycemic Effects of Novel *Panax notoginseng* Polysaccharide in Mice with Diet-Induced Obesity

**DOI:** 10.3390/foods11193101

**Published:** 2022-10-05

**Authors:** Xue Li, Hao Liu, Hui-Rong Yang, Ying-Jie Zeng

**Affiliations:** College of Food Science & Technology, Southwest Minzu University, Chengdu 610041, China

**Keywords:** *Panax notoginseng*, polysaccharide, structure, hypoglycemic effects

## Abstract

In the study, the structural features and hypoglycemic effects of a polysaccharide Pan from the root of *Panax notoginseng* were investigated. The molecular weight of Pan was 8.27 kDa. Structural analysis indicated that Pan mainly consisted of →3)-β-L-Rha*p*-(1→, →3,6)-β-D-Gal*f*-(1→ and →6)-β-D-Gal*f*-(1→ residues with acetyl groups. Pan exhibited good antioxidant activity. Pan could slow down the body weight and the content of blood glucose in the high-fat diet-induced mice, further suppress GLUT-2 and SGLT-1 expression in the intestines, and enhance p-IRS and p-AMPK expression in the livers, finally exhibiting hypoglycemic effects. The results could supply a direction for further research on polysaccharides as components for the control of hyperglycemia induced by obesity and diabetes.

## 1. Introduction

Type 2 diabetes mellitus (T2DM), which is induced by genetic and environmental factors, is a health issue of global concern [1]. As known to us, obesity is the main inducement resulting in hypertension, T2DM and other diseases [2]. Furthermore, the number of people who suffer from T2DM will rapidly grow to 642 million by the year 2040. Therefore, excavating effective coping strategies is extremely urgent to protect against T2DM. More and more people are coming to the agreement that healthy and balanced diets are the economical, practical and efficient mode to protect us from obesity and T2DM [3]. In recent years, increasingly natural products have been confirmed to effectively alleviate obesity and DM, and have been further developed as functional food and dietary supplements [4,5,6,7].

*Panax notoginseng* (Burkill) F.H.Chen ex C.Y. Wu & K.M. Feng is a rare traditional medicinal plant that is widely distributed in the southwest of China, Burma and Nepal [8]. Moreover, *P. notoginseng* has been used for the treatment of cardiovascular diseases, diabetes and inflammation [9,10,11]. There are some studies about polysaccharides from *P. notoginseng*; however, only a few studies from the literature reveal their structure–function relationship. As the important biological macromolecules from natural sources, polysaccharides usually exhibit immunological, antitumor and prebiotic effects, and hepatoprotective, hypolipidemic and antioxidant activities [12,13,14].

Therefore, in this study, a polysaccharide from *P. notoginseng* (Pan) was extracted, purified and characterized, and the hypoglycemic effects of Pan were investigated on high-fat diet (HFD)-induced mice. The study contributes to developing Pan as a functional food and dietary supplement for the purpose of preventing obesity and diabetes.

## 2. Materials and Methods

### 2.1. Materials and Chemicals

The analytical grade reagents were from Gibco (Grand Island, NY, USA), Santa Cruz Biotechnology (Santa Cruz, CA, USA) and Cell Signaling Technology (Boston, MA, USA). All other analytical reagents were from China. Chengdu Dossy Experimental Animals Co., Ltd. (Chengdu, China) supplied the normal diets and HFDs (60% fat, 20% carbohydrates and 20% protein).

### 2.2. Extraction and Purification of P. notoginseng Polysaccharides

The root powder from *P. notoginseng* was extracted via boiling water with the ratio of material to liquid of 1:25 (*m*/*v*) for three times, and each time kept for 4 h. After concentration and centrifugation, the supernatant was treated with 95% ethanol (*v*/*v*) at 4 °C for 12 h. The precipitate was collected and dried at 60 °C for 8 h. The dried compounds were the crude polysaccharides of *P. notoginseng*.

The protein and pigment in crude polysaccharides were removed via the AB-8 resin and Sevag reagent. Subsequently, the DEAE-52 chromatography column and Sephadex G-200 column were applied to purify the polysaccharides according to the published study [15].

### 2.3. Structural Characteristics Analysis

The sugar content of the *P. notoginseng* polysaccharide was detected according to the phenol-sulfuric acid method.

The monosaccharide composition and methylation of polysaccharides were assessed based on the published study [16], whereas the uronic acid contents such as galacturonic acid (GalA) and glucuronic acid (GlcA) were determined via ion chromatography (IC) [17]. The molecular weight was performed on an high performance gel permeation chromatography (HPGPC) system according to a previous report [18]. The structural features of the polysaccharide was evaluated via nuclear magnetic resonance (NMR) spectroscopy [19].

### 2.4. Antioxidant Activity Analysis

The scavenging capacity of the DPPH free radical in the *P. notoginseng* polysaccharide was evaluated according to a previous report [20], the scavenging capacity of the ABTS free radical in the *P. notoginseng* polysaccharide was evaluated according to the description of You et al. [21], and the ORAC value of the *P. notoginseng* polysaccharide was determined by Huang et al. [22].

### 2.5. MTT Assay

The cytotoxicity of the *P. notoginseng* polysaccharide was detected by the intestinal-epithelial Caco-2 cell line. The cells incubated in 96-well plates (each hole with 5 × 10^3^ cells, 0.2 mL) were treated with phosphate buffered saline (PBS) and 50, 100, 150 and 200 μg/mL of polysaccharide samples at 37 °C for 24 h; then, the cells were incubated with 3-(4,5)-dimethylthiahiazo (-z-y1)-3,5-di- phenytetrazoliumromide (MTT) for another 4 h. Finally, the cell precipitates were collected and dissolved in 150 μL of dimethyl sulfoxide (DMSO). The data were figured as follows:

$$\mathrm{Cell}\mathrm{viability}=\frac{OD492_{Experimental\;group}}{OD492_{PBS-treated\;group}}$$

### 2.6. Animals and Treatments

Female C57BL/6 mice (8 weeks old, 19.2 ± 0.5 g) were purchased from the Chengdu Dossy Experimental Animals CO., LTD. (Chengdu, China), and all animal protocols were approved by the Southwest Minzu University Center for Animal Experiment/Animal Biosafety Laboratory (Chengdu, China). Based on the previous method [23], after acclimation for one week, the mice were randomly divided into 5 groups: the normal diet (ND) group, the high-fat (HF) group, and the groups supplied with 50, 100 and 200 μg/mL of polysaccharide samples. 

Only the contents of the fasting blood glucose (FBG) were detected every week after 1 week of the treatments. The other sample collections were carried out according to the operations of Li et al. [23] at the end of the 7th week.

The contents of the triglycerides (TGs), total cholesterol (TC), low-density lipoprotein cholesterol (LDL-C) and high-density lipoprotein cholesterol (HDL-C) were evaluated via the corresponding assay kits based on the manufacturer’s descriptions (Jiancheng, Nanjing, China). The levels of serum HbA1c and insulin were determined via enzyme-linked immunosorbent assay (ELISA) kits (JianglaiBio, Shanghai, China).

### 2.7. Oral Glucose Tolerance Test (OGTT)

The OGTT evaluation was carried out based on a previous report with some modifications [24]. The female C57BL/6 mice were administered with 50, 100 and 200 μg/mL of polysaccharide samples and an equal volume of water (CT groups, 10 mice/group), at 50 mg/kg of body weight (BW). Thirty minutes later, the mice were administered 15% glucose at 1.5 g/kg of BW. The glucose levels were determined via the medical blood glucose meter (On Call Plus REF G113-232, Acon Biotech (Hangzhou) Co., Ltd., Hangzhou, China) at 0, 15, 30, 60, 90 and 120 min after the glucose administration.

### 2.8. Western Blot

The Western blot experiment was conducted according to the previous literature [25] with some modifications.

### 2.9. Statistical Analysis

All of the data are expressed as means ± standard deviations (SD). The statistical analysis was performed using a one-way ANOVA with Duncan’s multiple comparison tests among the groups. Statistical significance was considered at *p* < 0.05.

## 3. Results and Discussion

### 3.1. Molecular Weight, Monosaccharide Composition and Uronic Acid Levels

**Figure 1:**
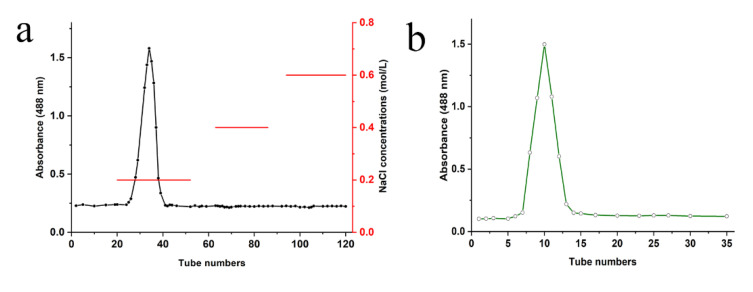
Chromatography of Pan purified via DEAE-52 chromatography (**a**) and Sephadex G-200 (**b**).

**Figure 2:**
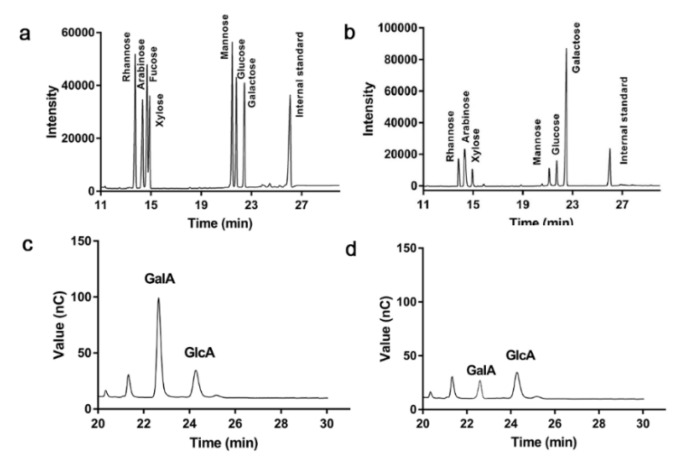
GC of standard monosaccharides (**a**) and Pan (**b**); IC of uronic acid standard (**c**) and Pan (**d**).

The polysaccharide Pan was obtained via DEAE-52 (Figure 1a) and Sephadex G-200; only a single elution peak appearing in the elution curve (Figure 1b), which suggested that Pan was homogeneous. The contents of sugar and protein in Pan were 91.39% and 1.14%, respectively. The molecular weight of Pan was 8.27 kDa. The results showed that Pan consisted of rhamnose (Rha), arabinose (Ara), xylose (Xyl), mannose (Man), glucose (Glc) and galactose (Gal) at a molar ratio of 11.7:23.3:3.27:4.7:6.4:32.6, respectively (Figure 2a,b). Meanwhile, the uronic acid of GlcA and GalA existed in Pan at a molar ratio of 2.64:4.03 (Figure 2c,d).

### 3.2. Methylation Analysis

According to Table 1, the main glycosidic linkages in Pan were 2,3,4-Me_3_-Rha*p*, 3-Me-Rha*p*, 2,3,5-Me_3_-Ara*f*, 2,3-Me_2_-Ara*f*, 2-Me-Ara*f*, 2,3-Me_2_-Xyl*p*, 2,4-Me_2_-Man*p*, 2,3,4,6-Me_4_-Glc*p*, 2,3,4-Me_3_-Glc*p*, 2,4-Me_2_-Glc*p*, 2,4,6-Me_3_-Glc*p*, 2,3,4,6-Me_4_-Gal*p*, 2,3,6-Me_3_-Gal*p*, 2,3,4-Me_3_-Gal*p*, and 2,3-Me_3_-Gal*p* with different molar percentages. The results suggested that Pan exhibited multiple backbones consisting of different glycosidic linkages. Furthermore, the galactose and rhamnose had the highest Pan derivatives.

### 3.3. NMR Spectroscopy Assay

**Figure 3:**
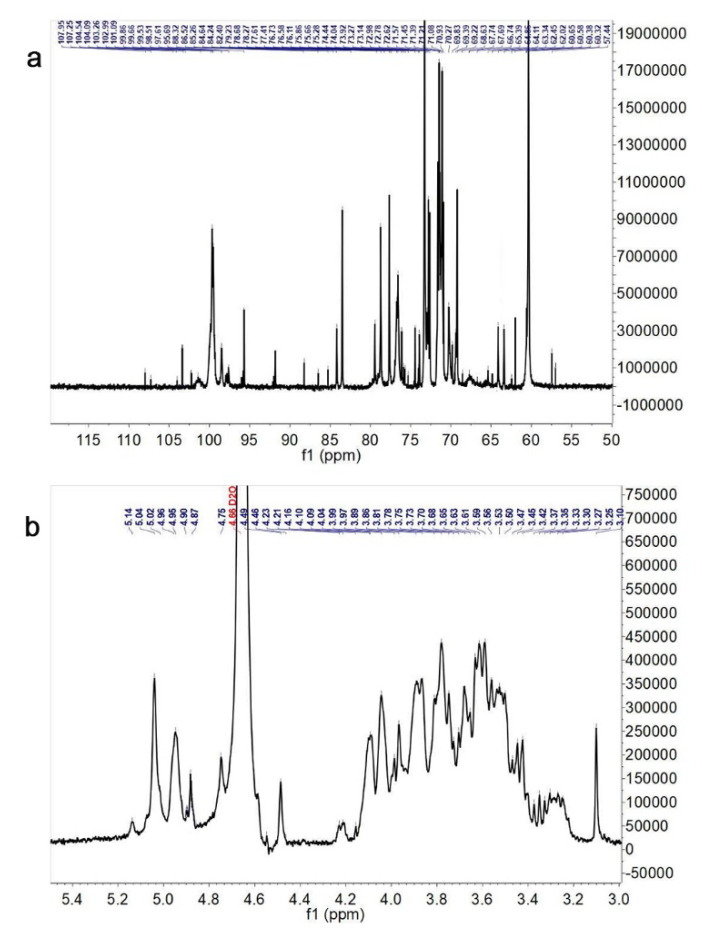
^13^C (**a**) and ^1^H (**b**) NMR spectrum of Pan.

The further structure elucidation of Pan was performed by using the ^13^C and ^1^H NMR spectra (Figure 3a,b). The typical signals belonging to polysaccharides were presented in ^1^H and ^13^C NMR at the chemical shifts of δ H 3.0~5.5 ppm and δ C 60~110 ppm for Pan. The anomeric protons of the polysaccharide are generally located at δH 3.0~5.5 ppm in the 1H NMR spectra, and the signals from δ 3.0 to δ 4.3 ppm in the ^1^H NMR spectrum were assigned to these protons from C2~C6 of Pan, which were attributed based on the reported literature [18,26,27]. The signals of 60~80 ppm in the ^13^C NMR spectrum represented the typical carbohydrate absorption peaks. Based on the published literature [28], the δ H 5~6 ppm region was mainly assigned to the α-anomeric protons, whereas a large percentage of the β-anomeric protons were located at the δ H 4~5 ppm.

Based the wide chemical shift dispersion in the ^13^C NMR spectrum (Figure 3a), the anomeric carbon signals were found at δ 60~108 ppm, and the corresponding ^13^C shifts were at δ 107.53, 107.45, 103.17, 101.45, 100.73 and 97.37 ppm. The β-glucoside hetero-carbon signals were located from δ 101 to 110 ppm, whereas the chemical shifts from δ 5.0 to 5.4 ppm were attributed to the anomeric proton. The anomeric proton resonance signals (δ 5.18, 5.00, 5.03, 4.45 and 5.00 ppm) and anomeric carbon resonance signals (δ 107.53, 93.37, 107.45, 100.73, 101.45 and 103.17 ppm) conformed with H-1 and C-1 of eleven anomeric residues: →5)-α-L-Araf-(1→, →3,6)-α-L-Manp-(1→, →3)-β-L-Rhap-(1→, α-D-Glcp-(1→, →4)-β-D-Glcp-(1→, →3,6)-β-D-Galf-(1→, and →6)-β-D-Galf-(1→, respectively.

Based on the above structure analysis, Pan possessed different glycosidic linkages in previous reports [9,10,18]. Chan et al. [9] found that the polysaccharides PNPA-1A and PNPA-2A were primary composed of 1,4-β-D-galactans, 1,5-α-L-arabinan and arabinogalactan II (AG-II); PNPA-3A was mainly made up of the rhamnogalacturonan I (RG-I)-type pectin with the side chains of 1,4-β-D-galactan and 1,5/1,3,5-α-L-arabinan; PNPA-1B, PNPA-2B and PNPA3B majorly consisted of homo-galacturonan (HG) and were accompanied with some RG-I and rhamnogalacturonan II (RG-II). Liu et al. [10] reported that a polysaccharide obtained via a gradient elution from the residue of *P. notoginseng* was mainly composed of →4)-α-D-GalA*p*-(1→4-β-L-Rha*p*-1→4)-β-D-Gal*p*-(1→ with the side chains α-L-Ara*f*-1→5)-α-L-Ara*f*-(1→ connecting to the backbone at O-3 of →4-β-L-Rha*p*-1. Feng et al. [18] had found that a novel polysaccharide MRP5, obtained from the root of *P. notoginseng* using the column chromatography method, was composed of →3)-β-Glc*p*-(1→, →6)-β-Glc*p*-(1→, →3, 6)-Glc*p*-(1→, β-Glc*p*-(1→, →3)-β-Gal*p*-(1→, →3, 6)-β-Gal*p*-(1→, →3)-α-Rha*p*-(1→, →3)-α-Ara*f*-(1→, and α-Ara*f*-(1→ residues. According to the above literature, one hypothesis could be drawn that the extraction method might cause an important effect on the structure features of polysaccharides from the same host. A further study remains to be carried out to reveal the exact impact of the extraction method on the structure of polysaccharides.

### 3.4. Antioxidant Activity Assay

**Figure 4:**
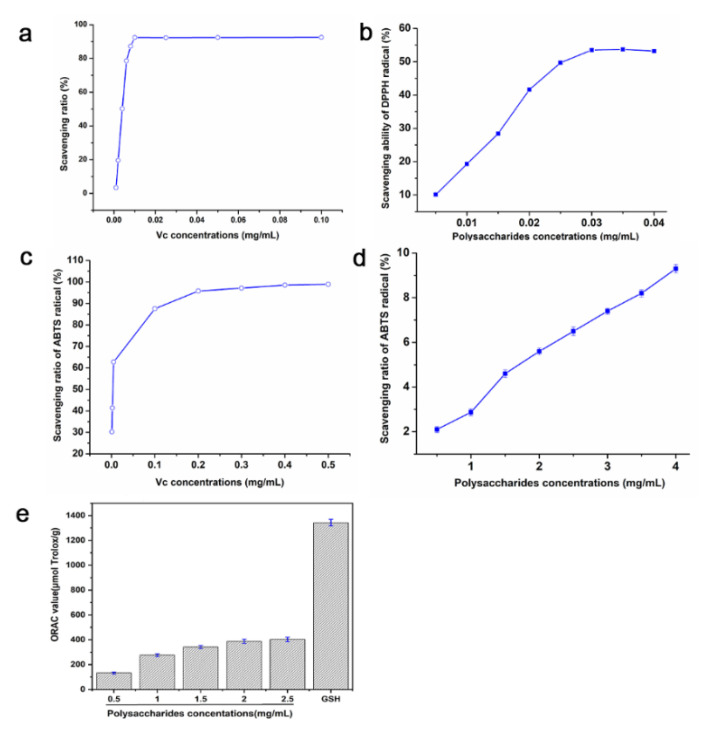
DPPH radical scavenging effects of Vc (**a**) and Pan (**b**); ABTS radical scavenging effects of Vc (**c**) and Pan (**d**); ORAC free radical scavenging via GSH and different concentrations of Pan (**e**).

The DPPH radical scavenging capabilities of Pan and Vc (positive group) were presented in Figure 4a,b. The DPPH radical scavenging ratio increased with the concentrations of polysaccharides and Vc. When the contents of Pan were 0.035 and 0.03 mg/mL, the scavenging ratios of the DPPH radical were 60.7% and 53.5%, respectively, which were lower than 93.4% of 0.01 mg/mL of Vc. The IC_50_ value of the DPPH radical scavenging ratio of Pan was 0.028 mg/mL, which was higher than the IC_50_ of Vc (about 0.005 mg/mL). In addition, compared with the positive group Vc, Pan also possessed some level of ABTS radical scavenging capability with the increase in the concentrations (Figure 4c,d), and there was still a huge gap compared with Vc. Furthermore, the ABTS scavenging capability of Pan presented a dose-dependent effect.

The ORAC of Pan at 2.5 mg/mL was 375.29 ± 13.8 μM Trolox/g (Figure 4e), which was equivalent to 1/4 of the GSH’s ORAC value (1343.14 ± 26.53 μM Trolox/g). Reactive oxygen species (ROS) inevitably caused oxidative damage to DNA and proteins in biological cells, and then induced other diseases [29]. Interestingly, the antioxidant activities of Pan were similar with the two novel polysaccharides MRP5 and MRP5A obtained from the root of *P. notogins*, reported by Feng et al. [18]. Therefore, the above results show that Pan could be used as a pure, natural antioxidant to replace some commonly used but toxic antioxidants.

### 3.5. Cytotoxicity Assay

**Figure 5:**
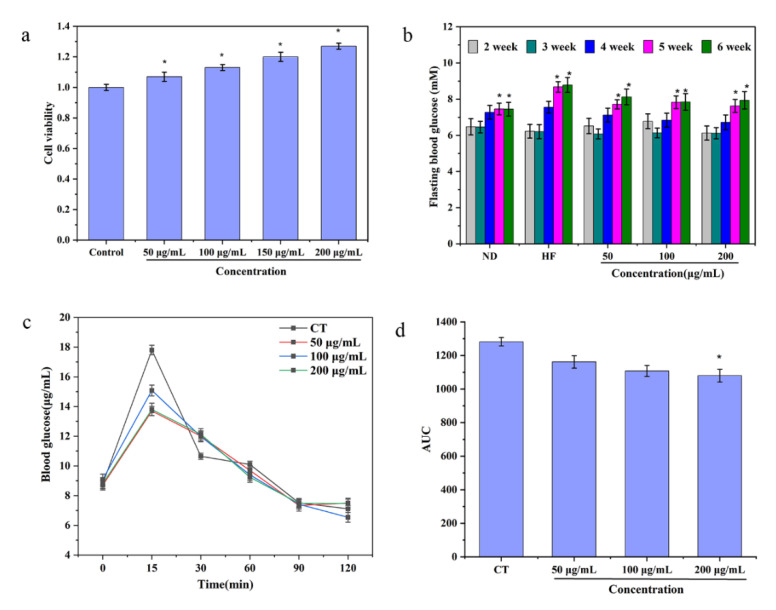
(**a**) The cytotoxicity effect of Pan on Caco-2 cells. Groups bearing * differ significantly at *p* < 0.05 with the control group (PBS-treated cells). (**b**) The levels of FBG in mice after 6 h fasts being fed normal diets (ND group), HFDs (HF group), and HFDs supplemented with 50 msg/kg/d Pan (50, 100 and 200 μg/mL treatment groups). Groups within the same week bearing * differ significantly at *p* < 0.05. (**c**) Suppression of blood glucose levels in OGTT by different concentrations of Pan. (**d**) Areas under the curves (AUC). Groups bearing * differ significantly at *p* < 0.05 against the CT group.

It was necessary to evaluate the cytotoxicity of Pan using an MTT test. As shown in Figure 5, it was obvious that when the contents of Pan were 50, 100, 150 and 200 μg/mL, there was no cytotoxicity in the Caco-2 cells, indicating that Pan can be safely used for future animal experiments.

### 3.6. Variation in Mice Hyperglycemia

Mice with high-fat diets (HFDs) could appear obese, hyperglycemic, hyper-insulinemic, insulin resistant, glucose intolerant and so on [30]. Therefore, to ascertain the hypoglycemic activity of Pan in vivo, the mice were administered with three concentrations of Pan (50 μg/mL, the lowest dosage group; 100 μg/mL, the median dosage group; 200 μg/mL, the highest dosage group). After administration for 5 weeks, the concentration of FBG in the HF group presented a significant increase and was much higher than that of the ND group, which indicated that the model of hyperglycemia had been successfully built. Moreover, Pan with different concentrations could effectively lower the fasting hyperglycemia caused due to the HFD. It was interesting to find that the administration of 100 μg/mL and 200 μg/mL of Pan was more beneficial to reduce the contents of FBG than the group treated with 50 μg/mL of Pan after 6 weeks (Figure 5b). It had been confirmed that the HbA1c exhibited some unique characteristics such as high stability, accuracy and precise reflection of chronic glycemic levels; rhus, it could be considered as the essence index to accurately diagnose T2DM [31,32]. Therefore, the contents of HbA1c were determined. The results showed that all three groups could significantly decrease the HbA1c levels compared with the HF group after the seven weeks of treatment, which suggested that there were hypoglycemic effects of Pan on the HFD-induced obese mice.

The postprandial hyperglycemia regulation is also an indicator that reflected the hypoglycemic effect on FBG. The OGTT results indicated that the 50 μg/mL and 100 μg/mL of Pan effectively reduced the postprandial hyperglycemia levels, but the postprandial hyperglycemia levels of the 200 μg/mL Pan-treated group could be significantly lowered down compared with the other groups (Figure 5c,d).

In a word, Pan could efficiently alleviate the contents of FBG and serum HbA1c in HFD-induced obese mice; meanwhile, it also lowered the concentrations of postprandial blood glucose, demonstrating that Pan actually has the capacity for lowering hyperglycemia in HFD-induced obese mice.

As shown in Table S1, there were no obvious effects of Pan on the food intakes and the contents of HDL-C, LDL-C, TGs, and insulin, except that Pan decreased the concentration of TC.

**Figure 6:**
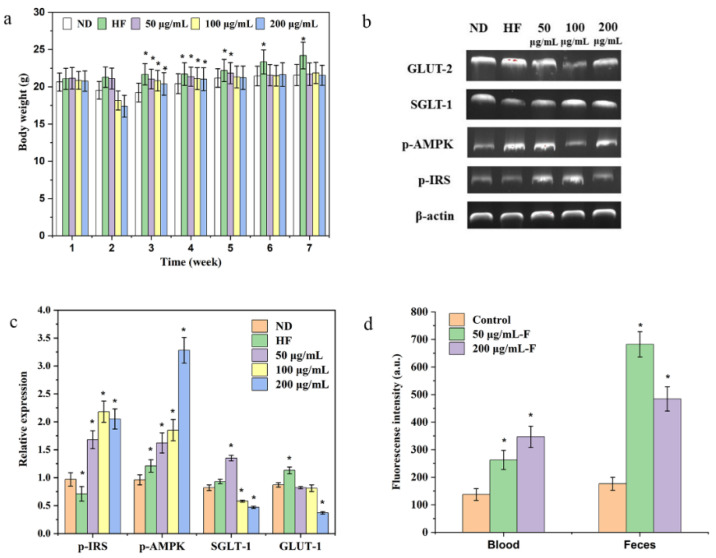
(**a**) Impacts of Pan (50 mg/kg/d) on the BWs of mice. (**b**,**c**) The expressions of SGLT-1 and GLUT-2 in the intestines and the expressions of p-IRS and p-AMPK in the livers of mice orally given 50, 100 and 200 μg/mL of Pan for 7 weeks. (**d**) Fluorescence intensities of FITC-labeled Pan (50 μg/mL-F and 200 μg/mL-F) in blood/feces. The asterisk (*) indicates the significant differences at *p* < 0.05 against the ND group or control group.

The effects of Pan on the BW of mice were also measured. The results showed that all three concentrations of Pan could reduce the BW after the third week of treatment in contrast to the HF group (Figure 6a). The reduction functions of the highest- and medium-level treatment groups preceded the lowest-level treatment group.

### 3.7. The Underlying Hypoglycemic Mechanism of Pan

Based on the hypothesis that the intake of Pan would lessen the levels of glucose in the blood circulation transported from the gastrointestinal tract, the major glucose transport proteins SGLT-1 and GLUT-2 in the intestines were collected, and then, the Western blot technique was adopted to measure the above two transport proteins’ expression, which transported glucose from the intestine into the blood [33]. As shown in Figure 6b,c, the 100 μg/mL and 200 μg/mL of Pan significantly inhibited the expressions of SGLT-1 and GLUT-2 in contrast to the HF group.

As a primary metabolic organ maintaining the glucose homeostasis within the body, the liver was strongly linked with the blood glucose metabolism. As shown in Figure 6b,c, the relative expressions of p-IRS and p-AMPK in the liver of HFD-induced obese mice were significantly increased in contrast to the HF group, and especially the 100 μg/mL and 200 μg/mL of Pan presented remarkable enhancement of the p-IRS and p-AMPK expressions.

Based on the above results, Pan caused hypoglycemic effects on the HFD-induced obese mice. Furthermore, the hypoglycemic mechanism of Pan might be attributed as follows: Firstly, Pan downregulated the expression of the primary glucose transporters GLUT-2 and SGLT-1 (Figure 6b,c), because SGLT-1 participated in regulating the transports of the intestinal glucose, and if the SGLT-1 was subjected to being suppressed, the hypoglycemic impacts of indigestible carbohydrates would be influenced [34,35]. Meanwhile, due to the decrease in blood glucose levels and BW from 100 μg/mL and 200 μg/mL of Pan, it could be inferred that the decrease in blood glucose resulted from the suppression of the GLUT-2 and SGLT-1 expression. Secondly, Pan enhanced the p-IRS and p-AMPK expression in the livers of HFD-induced obese mice (Figure 6b,c). The important function of tyrosine phosphorylation on IRS (Tyr-p-IRS) was regulating the insulin dependence of glucose and lipid metabolism via the upregulation of the recognition capability of the insulin receptors to insulin. It was reported that the decrease in Tyr-p-IRS was strongly linked with the insulin resistance of diabetes and obesity [36,37,38]. Hence, the increase in Tyr-p-IRS indicated that the hypoglycemic activity of Pan was closely related to the signaling improvement of the hepatic insulin-signaling pathway in the HFD-induced obese mice. As the primary intracellular energy sensor and the master switch of metabolism, AMPK played the crucial role in maintaining glucose homeostasis [39,40]. There were two pathways for the phosphorylation of AMPK to decrease the contents of blood glucose: one way was to enhance the uptake of glucose and another way was to downregulate the expressions of hepatic gluconeogenic gene in the liver. A previous report had found that several carbohydrates were able to activate the AMPK pathway to prevent hepatic cells from the high-glucose-induced damage, and the AMPK pathway could be considered as the therapeutic target for diabetes [41]. Therefore, the major reason for hyperglycemia decreasing with 50, 100 and 200 μg/mL of Pan was attributed to the p-AMPK signal pathway.

## 4. Conclusions

In this study, the structure and hypoglycemic activity of Pan were explored. The Mw, monosaccharide compositions and glycosidic linkages of Pan were revealed. Meanwhile, the hypoglycemic effects of Pan were attributed as follows: firstly, Pan downregulated the primary glucose transporters GLUT-2 and SGLT-1 and their expression; secondly, Pan up-regulated the p-IRS and p-AMPK expression in the liver of HFD-induced obese mice; finally, the AMPK pathway was activated to prevent hepatic cells from experiencing high-glucose-induced damage. The study obtained useful information to help Pan be developed as a functional food and dietary supplement candidate for the purpose of controlling obesity and DM.

## Figures and Tables

**Table 1:** Methylation analysis results of Pan.

Methylated Sugars	Linkage Types	Molar Ratio (%)
2,4-Me_2_-Man*p*	1,3,6-linked-Man*p*	4.6
2,3,4-Me_3_-Rha*p*	1-linked-Rha*p*	12.03
3-Me-Rha*p*	1,2,4-linked-Rha*p*	8.25
2,3,4,6-Me_4_-Glc*p*	1-linked-Glc*p*	29.45
2,3,4-Me_3_-Glc*p*	1,6-linked-Glc*p*	111.89
2,4-Me_2_-Glc*p*	1,3,6-linked-Glc*p*	46.12
2,4,6-Me_3_-Glc*p*	1,3-linked-Glc*p*	2.56
2,3-Me_2_-Xyl*p*	1,4-linked-Xyl*p*	17.82
2,3,4,6-Me_4_-Gal*p*	1-linked-Gal*p*	39.36
2,3,6-Me_3_-Gal*p*	1,4-linked-Gal*p*	16.67
2,3-Me_3_-Gal*p*	1,4,6-linked-Gal*p*	32.55
2,3,5-Me_3_-Ara*f*	1-linked-Ara*f*	23
2,3-Me_2_-Ara*f*	1,5-linked-Ara*f*	33.97
2-Me-Ara*f*	1,3,5-linked-Ara*f*	11.68

## Data Availability

Data are contained within the article or Supplementary Materials.

## Supplementary Materials

The following supporting information can be downloaded at: https://www.mdpi.com/article/10.3390/foods11193101/s1, Table S1. Food intakes and the contents of LDL-C, HDL-C, TGs, TC, insulin and HbA1c in the HFD-induced obese mice.
