# Supplementary material for: Hypoglycemic Effects of Novel Panax notoginseng Polysaccharide in Mice with Diet-Induced Obesity
Source: Foods. 2022 Oct 5;11(19):3101. doi: 10.3390/foods11193101 (PMC9562645; doi:10.3390/foods11193101)
Supplement: Supplementary file 1 [file foods-11-03101-s001.zip › foods-1899414-supplementary.pdf]

Supplementary Materials

Hypoglycemic Effects of Novel *Panax notoginseng* Polysaccharide in Mice with Diet-Induced Obesity

Table S1. Food intakes and the contents of LDL-C, HDL-C, TG, TC, insulin, and HbA1c in the HFD-induced obese mice.

Groups	Food in-take (g/kg/d)	LDL-C (μM)	HDL-C (mM)	TG (mM)	TC (mM)	Insulin (mM)	HbA1c (mM)
ND	182±27	0.26±0.06	3.82±0.23	1.28±0.26	3.14±0.32	17.8±1.57	172.34±15.38
HF	112±18	0.31±0.04	4.92±0.42	1.58±0.06	4.32±0.22	19.53±2.18	218.22±30.21
50 μg/mL	108±19	0.30±0.05	4.52±0.23	1.72±0.09	3.41±0.32	18.26±3.12	163.52±12.59
100 μg/mL	118±21	0.32±0.06	4.61±0.27	1.91±0.46	3.27±0.26	18.64±1.94	181.47±19.63
200 μg/mL	107±22	0.30±0.12	4.91±0.32	1.68±0.16	3.51±0.18	17.52±1.37	171.43±15.29
